# Jak2 Is a Negative Regulator of Ubiquitin-Dependent Endocytosis of the Growth Hormone Receptor

**DOI:** 10.1371/journal.pone.0014676

**Published:** 2011-02-09

**Authors:** Joyce Putters, Ana C. da Silva Almeida, Peter van Kerkhof, Agnes G. S. H. van Rossum, Ana Gracanin, Ger J. Strous

**Affiliations:** 1 Department of Cell Biology and Institute of Biomembranes, University Medical Center Utrecht, Utrecht, The Netherlands; 2 Drug Discovery Factory BV, Bussum, The Netherlands; 3 Department of Clinical Sciences of Companion Animals, Faculty of Veterinary Medicine, Utrecht University, Utrecht, The Netherlands.; University of Geneva, Switzerland

## Abstract

**Background:**

Length and intensity of signal transduction via cytokine receptors is precisely regulated. Degradation of certain cytokine receptors is mediated by the ubiquitin ligase SCF(βTrCP). In several instances, Janus kinase (Jak) family members can stabilise their cognate cytokine receptors at the cell surface.

**Principal Findings:**

In this study we show in Hek293 cells that Jak2 binding to the growth hormone receptor prevents endocytosis in a non-catalytic manner. Following receptor activation, the detachment of phosphorylated Jak2 induces down-regulation of the growth hormone receptor by SCF(βTrCP). Using γ2A human fibroblast cells we show that both growth hormone-induced and constitutive growth hormone receptor endocytosis depend on the same factors, strongly suggesting that the modes of endocytosis are identical. Different Jak2 RNA levels in HepG2, IM9 and Hek293 cells indicate the importance of cellular concentration on growth hormone receptor function. Both Jak2 and βTrCP bind to neighbouring linear motifs in the growth hormone receptor tail without the requirement of modifications, indicating that growth hormone sensitivity is regulated by the cellular level of non-committed Jak2.

**Conclusions/Significance:**

As signal transduction of many cytokine receptors depends on Jak2, the study suggests an integrative role of Jak2 in cytokine responses based on its enzyme activity as well as its stabilising properties towards the receptors.

## Introduction

In mammalian cells the Janus kinase (Jak) family of protein tyrosine kinases comprises Jak 1,2,3 and Tyk2. All Jak family members are widely expressed except for Jak3 that is restricted to cells of the hematopoietic system. Jak molecules associate non-covalently with a membrane-proximal region in cytoplasmic tails of cytokine receptors and play crucial roles in the initial steps of cytokine signalling [1], [2].

At the C-terminus, Jak family members contain the tyrosine kinase domain preceded by a pseudokinase domain. The N-terminal half of Jak comprises a postulated FERM domain (four-point-one, ezrin, radixin, moesin) that binds to cytokine receptors and a potential SH2 domain [3]. Specific mutations in the FERM domain inhibit cytokine receptor association and concomitantly abrogate Jaks' ability to respond to ligand binding. Binding of the FERM domain to receptors has also been proposed to assist in cell surface localisation of several cytokine receptors [4], [5], [6], [7], [8], [9].

Growth hormone (GH) is a multifunctional, clinically important cytokine hormone that acts through its type I cytokine receptor, the growth hormone receptor (GHR). The type I cytokine receptor family further includes the prolactin (Prl) receptor, the erythropoietin (Epo) receptor, and the thrombopoietin (Tpo) receptor [10], [11]. All cytokine receptors lack intrinsic kinase activity. Instead, a conserved proline-rich domain in their cytosolic tail, box-1, functions as a binding site for Jak family members. In the case of GHR, ligand binding results in the activation of Jak2 molecules [12] that in turn phosphorylate each other's tyrosine residues , the receptor's cytosolic tail and downstream signalling molecules [13]. Both liganded and unoccupied GHRs are endocytosed via clathrin-coated vesicles and subsequently transported via endosomes to lysosomes [14]. Previously, we have shown that both endocytosis and transport to lysosomes require an active ubiquitin conjugation system and a 10-amino acid motif (UbE-motif inside the conserved box-2 region) in the cytosolic tail of GHR [15]. The SCF(βTrCP) ligase drives endocytosis and degradation of the GHR by binding with its WD40 domain to the non-conventional UbE motif of GHR [16].

GHR signalling depends on relative rotation of the pre-dimerised receptor subunits aligning two Jak2 molecules upon GH binding [17], [18]. Recent studies show that the Jak family also plays non-catalytic roles in regulating the cellular localisation and trafficking of cytokine receptors [6], [19]. For the GHR, increased Jak2 expression levels increase the fraction of mature GHRs [9].

GHR endocytosis provides an important means to control GH sensitivity. Patients with cancer-induced wasting are relatively GH-insensitive and have low GH binding protein titers, indicative for rapid endocytosis of GHRs [20]. It is therefore important to understand the cellular mechanisms involved in the regulation of GHR endocytosis.

Here, we demonstrate that Jak2 binding specifically inhibits GHR endocytosis, independent of its kinase activity. Studies by the group of Ihle have shown that phosphorylation of tyrosine 119 of Jak2 abrogates the interaction of Jak2 with several cytokine receptors [21], [22], [23]. Based on this and our own experiments, we hypothesise that GH stimulation releases Jak2 from the GHR. Combining fractionation studies with bioluminescence resonance energy transfer (BRET), we show that Jak2 activation indeed results in release of phosphorylated Jak2 from the GHR, after which the GHR is allowed to internalise via SCF(βTrCP)-mediated endocytosis and to be degraded.

## Results

### Jak2 specifically inhibits GHR endocytosis independent of its kinase activity

**Figure 1:**
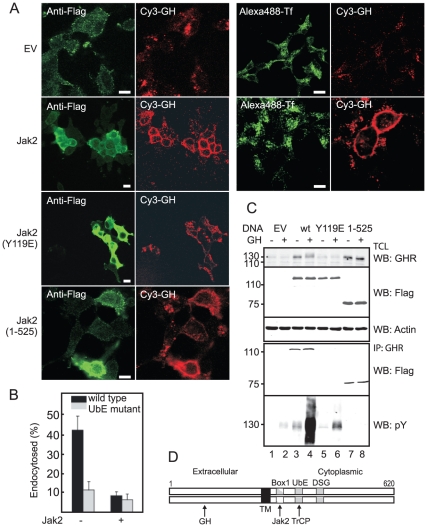
Jak2 inhibits GHR endocytosis. A. GHR-expressing Hek293 cells were transfected with empty vector (EV), Flag-Jak2, Flag-Jak2(Y119E) or Flag-Jak2(1-525). One coverslip was incubated for 15 min with Cy3-GH at 37°C, fixed and stained with anti-Flag (left panels). For EV- and Jak2-transfected cells, cells were also incubated with Cy3-GH and Alexa488-transferrin (Tf) for 15 min at 37°C (right panel). Bar, 5 µm. B. Hek293 GHR or GHR(UbE mutant)-expressing cells were transfected with Jak2 DNA or empty vector and, after 2 days, incubated for 2h on ice with 180 ng/ml ^125^I-GH. To measure uptake kinetics unbound label was removed and the cells were incubated at 37°C for 10 min. C. Hek293 cells were transfected as in 1A and treated with GH for 15 min at 37°C as indicated. Total cell lysates (TCL, upper panel) and GHR immunoprecipitations (lower panel) were analysed on western blot (WB) using anti-Flag, or anti-phosphotyrosine (pY). 130, mature GHR; 110, precursor GHR. Data are representative of three independent experiments. D. Diagram showing the different binding motifs in the dimerised GHR; Jak2 binds Box1, TrCP binds UbE). The transmembrane domains are the dimerisation domains.

Recently, we showed that the E3 ligase SCF(βTrCP) is required for GHR endocytosis via the UbE-motif of the GHR [16]. Other studies revealed that Jak2 stabilises the GHR [9], [24]. In Fig. 1D the different binding motifs are illustrated. To investigate whether Jak2 mediates GHR stabilisation by inhibiting GHR endocytosis, we expressed Jak2 in GHR-expressing Hek293 cells and incubated the cells with Cy3-GH to monitor GHR endocytosis. Cy3-GH uptake was strongly inhibited in cells expressing exogenous Jak2, while cells that did not express exogenous Jak2 contained less Cy3-GH, all of it being intracellular *en route to* the lysosomes (EV) (Fig. 1A). Exogenously expressed Jak2 did not interfere with clathrin-mediated endocytosis in general as is shown in Fig. 1A for transferrin, right panels, where we compared Cy3-GH and transferrin uptake. Hek293 cells that did not express GHR showed no Cy3-GH labelling (not shown), indicating that the entire fluorescent label originated from GHR activity. Thus, (over)expression of Jak2 causes a clear disruption of GH/GHR endocytosis.

To investigate whether the endocytosis inhibition depends on the interaction between GHR and Jak2, we took advantage of a study by Funakoshi-Tago and co-workers who showed that replacing a tyrosine residue with a glutamic acid at position 119 abrogates Jak2 binding to a subset of cytokine receptors, including the GHR [21]. Fig. 1A, lower panel, shows that expression of Jak2(Y119E) did not affect Cy3-GH uptake, indicating that Jak2-GHR interaction is needed for the inhibition of endocytosis of the GHR. We then asked whether Jak2 kinase activity is required. As Cy3-GH activates both GHR and Jak2, we transfected the cells with a DNA construct expressing only the N-terminal half of the molecule including FERM and SH2 domains, devoid of the (pseudo)kinase domains. As seen in Fig. 1A, Jak2(1-525) inhibited GHR endocytosis similar to wild-type Jak2. These data are in full agreement with work of Deng *et al.* who showed that binding of Jak2 is sufficient to inhibit GHR down-regulation [24]. To confirm that Jak2 indeed inhibits GHR endocytosis we determined the initial uptake rates of ^125^I-GH in cells co-transfected with GHR and Jak2. As seen in Fig. 1B, about 40% of ^125^I-GH was endocytosed within 10 min, whereas less than 10% endocytosed if Jak2 was co-expressed. This uptake rate is very similar if GH is bound to a GHR that lacks the βTrCP binding site (UbE motif). Co-expression of Jak2 had no significant additive effect on GHR endocytosis. Together, these results demonstrate that overexpression of Jak2 inhibits GHR endocytosis.

To further substantiate the results we expressed the various Jak2 species in the GHR-expressing cell line. Fig. 1C, upper panel, shows that both wild-type and Jak2(1-525) increased the steady state levels of several fold, dependent on the efficiency of transfection. The increase was solely due to decreased endocytosis of the mature (130kD) GHR, because the steady state levels of GHR in the endoplasmic reticulum (110kD) were unchanged. Notably, although expressed to the same level, the Jak2 binding mutant Jak2(Y119E) had little effect on the steady state of GHR. Incubation with GH slightly increased the apparent molecular weight of mature GHR in the presence of wild-type Jak2 but had no effect if the truncated form of Jak2 was co-expressed. Next, we analysed the Jak2-GHR interaction. As expected, both wild-type and truncated Jak2, but not Jak2(Y119E) co-immunoprecipitated with the GHR (Fig. 1C, panel 4). Exogenous Jak2(1-525) was unable to phosphorylate the GHR upon GH addition, while Jak2(Y119E) induced a very low level of phosphorylation as compared to wild-type Jak2, probably due to a low residual affinity of the mutant Jak2 for box-1 (lanes 4, 6, 8), indicating that Jak2-GHR binding is essential for GHR phosphorylation (Fig. 1C). Together, we conclude that Jak2 binding to the GHR inhibits GHR endocytosis independent of Jak2 kinase activity.

### Jak2 acts upstream of SCF(βTrCP)

**Figure 2:**
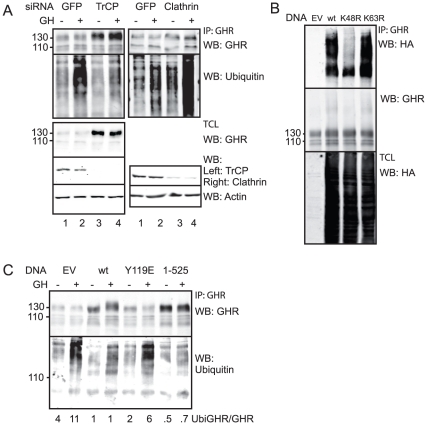
SCF(βTrCP) and Clathrin act downstream of Jak2. A. GHR-expressing Hek293 cells were either silenced for GFP (negative control), βTrCP or clathrin, and then treated for 15 min with GH at 37°C as indicated. GHR immunoprecipitations were analysed for ubiquitination. In the lower part of the figure the efficiency of silencing is visualised using anti-βTrCP or anti-Clathrin. B. Hek293 cells were transiently transfected with HA-tagged wild-type ubiquitin, ubiquitin K48R or ubiquitin K63R. Total cell lysates (TCL) and GHR immunoprecipitates were analysed on western blot using the indicated antibodies. EV, empty vector. C. Cells were transfected as in Fig. 1A and then treated for 15 min with GH at 37°C as indicated. GHR was immunoprecipitated from SDS-boiled lysates, and analysed for ubiquitination. 130, mature GHR, 110, precursor GHR. At the bottom of the figure the ratios of ubiquitinated versus 130kD-GHR are given. All data in this figure are representative of three independent experiments.

Based on our conclusion that Jak2 binding is sufficient to inhibit GHR endocytosis, we hypothesised that Jak2 must leave the GHR before the endocytosis machinery, including SCF(βTrCP) and clathrin, can proceed [16]. Although GHR does not seem to be an essential target for ubiquitination, the receptor becomes ubiquitinated during the process of endocytosis [25]. Here, we use GHR ubiquitination to measure progression of the GHR in the endocytosis process. In this scenario, it is expected that βTrCP gene silencing prevents GHR (K48) polyubiquitination, while clathrin silencing might accumulate (K48-ubiquitinated) GHR at the cell surface as it acts downstream of SCF(βTrCP). Dependent on the effectiveness, silencing of βTrCP caused a 5–10 fold accumulation of the mature (130kD) GHR at steady state, whereas its synthesis (110kD) remained unchanged. Upon βTrCP silencing, especially if GH was added to the cells, GHR ubiquitination was clearly inhibited (Fig. 2A). To ascertain that this polyubiquitin signal was K48-linked as is expected if SCF(βTrCP) is the ubiquitin ligase, we overexpressed wild-type and mutant ubiquitin that cannot make K48 and K63 polyubiquitin chains (K48R and K63R), respectively [26], [27], [28], [29]. As seen in Fig. 2B, ubiquitin K48R overexpression could not form poly-ubiquitin chains on GHR, whereas transfection of ubiquitin K63R had no effect. Thus, very likely βTrCP is involved in K48-linked ubiquitination of GHR during endocytosis.

Cargo selection into clathrin-coated pits is a general mechanism for cells to specifically transport membrane proteins into endosomes. Previously, we showed that GHR endocytosis occurs clathrin-mediated [30], [31]. In Fig. 2A, right panel, we show that silencing of clathrin indeed caused a very strong accumulation of ubiquitinated GHRs, especially if the cells were treated with GH. Quantification of the amounts of GHR is difficult due to variable and inefficient electrophoretic transfer of ubiquitinated proteins. To ascertain that the ubiquitin signals originate from GHR we used 1% boiling SDS buffers in all ubiquitination experiments to lyse the cells and to prevent (ubiquitinated) proteins to associate to GHR. We conclude that GHR ubiquitination occurs before endocytosis and depends on SCF(βTrCP) activity.

If Jak2 binding prevents the activity of SCF(βTrCP), exogenous Jak2 should accumulate GHR at the cell surface in a non-ubiquitinated state. Therefore, we overexpressed wild-type and mutant Jak2 in the GHR-expressing cell line and measured GHR ubiquitination. Upon wild-type Jak2 and Jak2(1-525) overexpression, GHR was stabilised, but GHR ubiquitination was decreased, demonstrating a strong inhibition of GHR ubiquitination (Fig. 2C, lane 4). To quantify the effects we measured the ratios of ubiquitinated GHR over total mature GHR: both in absence and presence of GH the ubiquitination of GHR was 4–10 times lower if either wild-type Jak2 or Jak2(1-525) was overexpressed compared to control cells (empty vector). As expected, Jak2(Y119E) showed GHR ubiquitination comparable to control cells. We conclude that Jak2 binding inhibits GHR ubiquitination and probably acts upstream of SCF(βTrCP) and clathrin.

### Phosphorylated Jak2 dissociates from the GHR

So far, we have shown that Jak2 inhibits GHR endocytosis non-catalytically. According to our hypothesis, Jak2 needs to detach from the receptor to allow endocytosis. As shown by Funakoshi-Tago *et al.*, phosphorylation of Y119 of Jak2 induces Jak2 dissociation of several cytokine receptors [21]. This implies that GH stimulation results in Jak2 release. As many studies have been performed with cell lines expressing exogenous GHR, large numbers of receptors relative to low numbers of (endogenous) Jak2 might disturb the control of endocytosis that is based on a stoichiometric relation between Jak2 and GHR.

**Figure 3:**
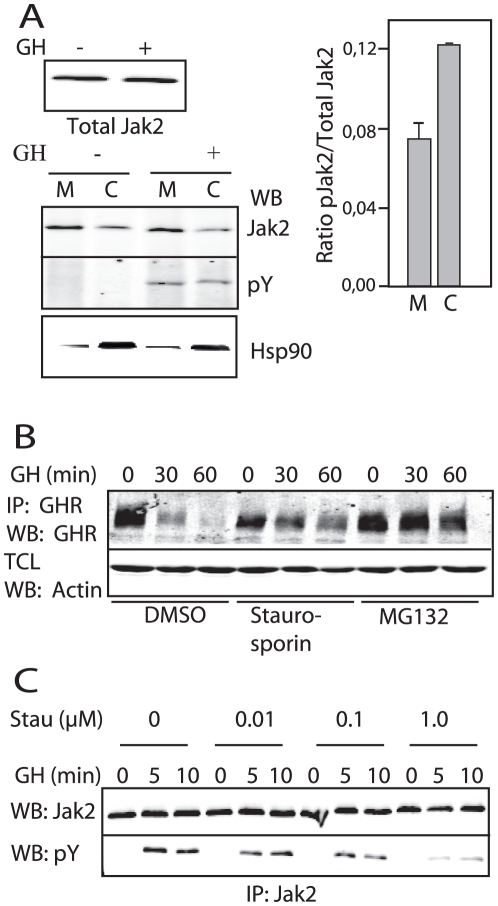
GH induces Jak2 phosphorylation and release from the GHR. A. IM9 cells were treated with GH for 1 h at 37°C as indicated, followed by cell fractionation. Membrane (M) and cytosol (C) fractions were immunoprecipitated for Jak2 and analysed with the indicated antibodies. The ratio of phosphorylated Jak2 (pJak2) and total Jak2 was calculated for each fraction. Total cell lysates were analysed for Hsp90, a cytosolic marker. B. IM9 cells were pretreated with vehicle (dimethylsulfoxide), staurosporin or MG132 for 1 h and stimulated with GH at 37°C as indicated. Total cell lysates (TCL) and GHR immunoprecipitations were analysed on western blot with the indicated antibodies. C. IM9 cells were pretreated with staurosporin (Stau) for 1 h followed by incubation with GH at 37°C as indicated. Jak2 immunoprecipitations were analysed with the indicated antibodies. Data in A, B, and C are representative of three independent experiments.

Human IM9 lymphoblasts have detectable levels of endogenous GHR and have been used in many cytokine receptor studies. An important property of this cell line is the fact that GHR is only endocytosed in presence of GH. If IM9 cells contain sufficient amounts of Jak2 to accumulate endogenous GHRs at the cell surface, we expect that GH stimulation triggers GHR endocytosis by Jak2 phosphorylation on Y119 and detachment from the GHR. Testing the endogenous Jak2-GHR interaction by co-immunoprecipitation failed, probably due to low efficiency of the antibodies. Therefore, we based our experiments on the findings of Behrmann *et al.*, who showed that Jak1 and −2 mainly occur membrane-bound, dependent on their ability to associate with cytokine receptors [32]. As Jak2 can only become phosphorylated if associated with a cytokine receptor [1], [33], we assume that phosphorylated Jak2, if present in the cytosol, has been released from GHRs. First, we established the relative distribution of total endogenous Jak2: 80% was membrane-bound (Fig. 3A, left lanes). No GHR was detectable in the cytosolic fraction (not shown).Treatment with GH did not change the overall Jak2 distribution (this is expected as Jak2 binds to many different substrates) but, the fraction of phosphorylated Jak2 remained almost 50% higher in the cytosol compared to that in the membrane fraction as measured over a period of 60 min (Fig. 3A, right lanes). These results indicate that phosphorylated Jak2 dissociates from the GHR upon GH stimulation. As it is unknown in what priority and proportion the different tyrosine residues in Jak2 are phosphorylated, it is difficult to assess why not all phosphorylated Jak2 is in the cytosol.

To ascertain that GH-induced GHR endocytosis (degradation) in IM9 cells depends on the ubiquitin system as previously shown for GHR endocytosis in various tissue culture cells, we used the proteasomal inhibitor MG132 [25]. Pre-treatment of IM9 cells with MG132 indeed completely inhibited GH-induced GHR degradation (Fig. 3B, lanes 7–9). To investigate whether GH-induced endocytosis depends on Jak2 activity, we inhibited endogenous Jak2 kinase activity with the kinase inhibitor staurosporin and treated the cells with GH. Previously, it has been shown that 1 µM staurosporin abolished GHR phosphorylation in CHO cells [34]. Fig. 3C shows the same result for Jak2 phosphorylation. In control cells GH induced complete degradation of the GHR within 60 min, while staurosporin treatment stabilised the mature GHR (Fig. 3B). The immunoprecipitations shown in Fig. 3B were performed after boiling in 2% SDS and reducing agents to exclude that the observed loss of GHRs resulted from a shift to the detergent-insoluble fraction. Whether or not the endocytosis occurred via transition through a detergent-insoluble stage as suggested by Goldsmith *et al.* [35], in any case the final result is an almost complete GH-induced degradation of GHR after 60 min. The results support the hypothesis that Jak2 kinase activity is required for GHR endocytosis. Although previous studies indicate an enhanced interaction between GHR and Jak2 upon GH stimulation [36], the conditions differ in timing and expression levels, indicating that the GHR-Jak2 interaction is dynamic during GH stimulation. This is also supported by the current view that signalling complexes preassemble at the plasma membrane [32], [37].

**Figure 4:**
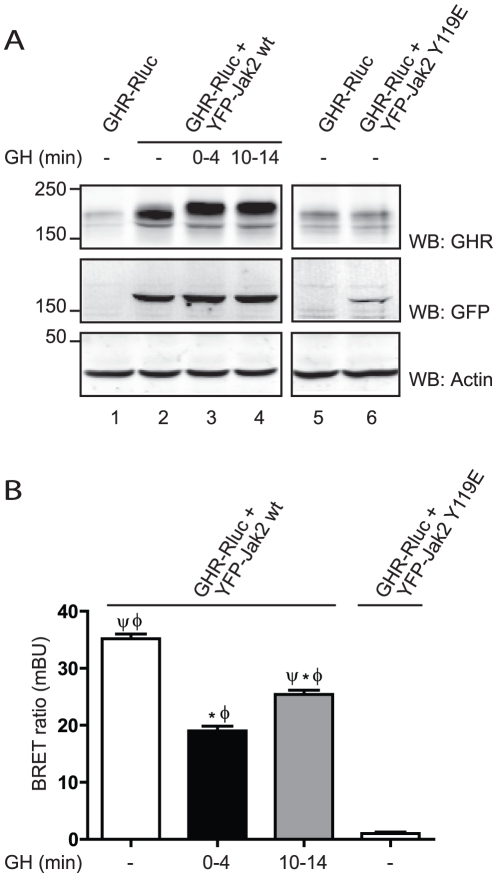
GH induces Jak2 release from GHR. Hek293-TR stable cell lines expressing GHR-Rluc and YFP-Jak2 (wt or Y119E) were seeded 24 h before the measurements; doxycycline was added in order to stimulate the expression of YFP-Jak2 constructs. BRET measurements were started immediately after GH addition (interval t = 0–4 min), or 10 min later (interval t = 10–14 min). A. Cells were lysed, and the proteins expression levels of the BRET pair in the cell lysates, were assessed by western blot, using the indicated antibodies. Data are representative for three independent experiments. Panel B shows the BRET ratios expressed in mBU, representative of 8 independent measurements ± S.E.M. (* significantly different from GHR-Rluc+YFP-Jak2 wt, minus GH condition, p<0.01; Φ significantly different from GHR-Rluc+YFP-Jak2 Y119E condition, p<0.01; Ψ significantly different from GHR-Rluc+YFP-Jak2 wt, plus GH [0–4 min] condition, p<0.01).

Resonance energy transfer techniques have recently emerged that allow the study of the dynamics of interactions of proteins in living cells [38], such as BRET. BRET has been extensively used to study a wide range of protein interactions, such as GPCRs [39], [40] and insulin receptor [41]. We have made use of this technology to study the effect of GH on the interaction between GHR and Jak2. We created and validated the interaction partners, Jak2, N-terminally fused to YFP (YFP-Jak2), and GHR, C-terminally fused to Renilla luciferase (GHR-Rluc). As a negative control, Jak2 Y119E, which does not bind GHR, was N-terminally fused to YFP (YFP-Jak2 Y119E). Hek293-TR cell lines constitutively expressing GHR-Rluc, as well as Jak2-YFP (wt or Y119E), under a doxycycline inducible promoter as described in the Methods section were used. In the absence of doxycycline, no acceptor protein (YFP-Jak2) is expressed, and, therefore, it is a condition of donor only (GHR-Rluc). Doxycycline induction resulted in the expression of YFP-Jak2 that (as expected) induced accumulation of GHR (compare lanes 2–4 with lane 1). Expression of the binding mutant, YFP-Jak2 Y119E, did not stabilise GHR-Rluc, (lanes 5 and 6). As seen in Fig. 4B BRET measurement detected a clear interaction between GHR-Rluc and YFP-Jak2, consistent with the association of Jak2 with the cytoplasmic tails of GHR in absence of GH. As predicted, no BRET signal could be detected in the cell line where YFP-Jak2 Y119E was expressed. Addition of GH, elicited a fast (time interval: 0–4 min) reduction of the BRET signal (of 46±6%) when compared to basal state. After 10 min the BRET ratio stabilised at the level of 65% (65±3%) of the basal signal. The short stimulation with GH was sufficient to cause a decreased migration of the mature GHR in the gel (Fig. 4A, lanes 3 and 4). Although it is important to realise that BRET cannot distinguish between a change in conformation that results in an increased distance between donor and acceptor, and dissociation events, our results are in agreement with a scenario where, at early time points after GH stimulation, Jak2 dissociates from the receptor due to its phosphorylation, but soon after it re-associates in a dynamic fashion, probably due to phosphorylation/dephosphorylation cycles. Since the Jak2 expression affects the levels of GHR, neither saturation nor competition assays could be performed. In addition, use of a kinase-inactive Jak2, that is unable to leave the GHR, would generate biased results due to unpredictable conformational changes in the BRET assay; therefore, no quantitative data regarding the interaction were obtained.

### Decreased Jak2 activity in coated pits

**Figure 5:**
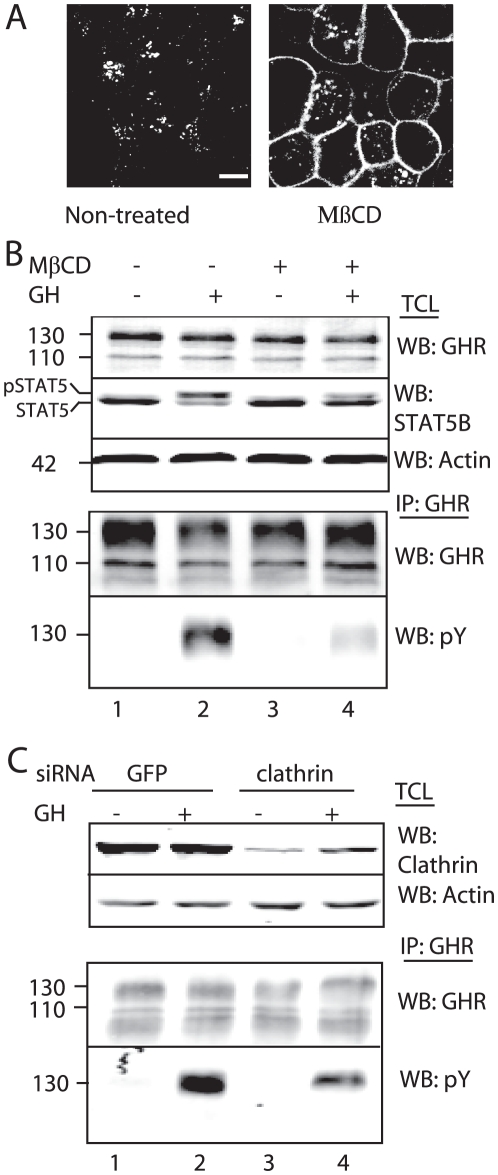
Decreased Jak2 activity in coated pit. A. GHR-expressing Hek293 cells were pretreated with MβCD for 1 h, incubated with Cy3-GH for 30 min at 37°C and the fluorescence was visualised with a confocal microscope. Bar, 5 µm. B. Cells were pretreated as in Fig. 4A, followed by incubation for 15 min with GH at 37°C as indicated. Total cell lysates (TCL, upper panel) and GHR immunoprecipitations (lower panel) were analysed on western blot with the indicated antibodies. 130, mature GHR; 110, precursor GHR. C. Cells were silenced either for clathrin or for GFP (as a negative control), followed by incubation for 15 min with GH at 37°C as indicated. Total cell lysates (TCL, upper panel) and GHR immunoprecipitations (IP, lower panel) were analysed on western blot with the indicated antibodies. 130, mature GHR; 110, precursor GHR. Data are representative for two independent experiments (B).

If Jak2 detaches after GH stimulation, we expect Jak2 activity to be absent in the GHR complexes that are about to be recruited into the coated pits. To test this we inhibited endocytosis at the coated pits by either methyl-β-cyclodextrin (MβCD) or by silencing clathrin. MβCD depletes cholesterol from the plasma membrane and inhibits clathrin-coated pit budding. This causes inhibition of internalisation of the transferrin receptor by 85% [42]. Fig. 5A shows inhibition of Cy3-GH uptake after treatment with MβCD as compared to non-treated cells. Next, we stimulated with GH and measured both GHR- and STAT5 phosphorylation. As seen in Fig. 5B, GHR phosphorylation was strongly inhibited in MβCD-treated cells as compared to non-treated cells that expressed comparable amounts of GHRs (lower panel, lanes 2 and 4). In addition, phosphorylation of STAT5, a major downstream signal transduction molecule, was decreased (upper panel, lanes 2 and 4). MβCD treatment did not affect the efficiency of GH-GHR interaction indicating a strong reduction of Jak2 activity at this stage of endocytosis. If clathrin was silenced, a 40% reduction of GHR phosphorylation was measured (Fig. 5C, lower panel). The reduction in GHR phosphorylation was not as prominent as for MβCD, probably due to the efficiency of gene silencing. These data support our hypothesis that GHRs that are about to be recruited by the endocytosis machinery can no longer be phosphorylated, suggesting the absence of Jak2 in the GHR complex. Although we measure a clear reduction of GHR phosphorylation under conditions of inhibited endocytosis with GH bound, an alternative explanation could be a failure of the GH-bound GHR to undergo the necessary conformational change to realign JAK2 monomers in the absence of cholesterol in the plasma membrane as well in the absence of a functioning clathrin mediated endocytosis pathway.

### GH-induced and constitutive endocytosis use the same endocytosis mechanism

**Figure 6:**
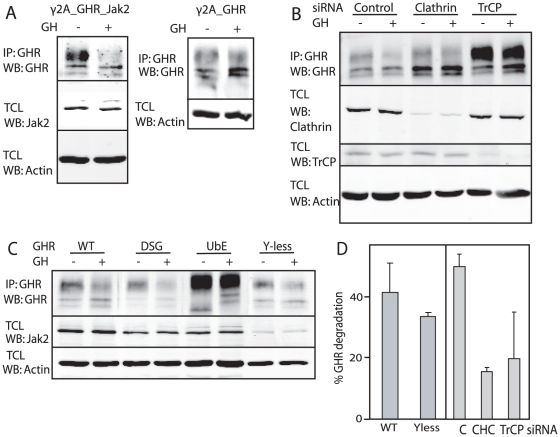
GH-induced and constitutive GHR endocytosis share the same characteristics. A. γ2A_GHR_Jak2 cells (left panel) and γ2A_GHR cells (right panel) were stimulated with 500 ng/ml GH for 90 min at 37°C as indicated. Total cell lysates (TCL) (lower panels) and GHR immunoprecipitates (upper panels) were analysed on western blot with the indicated antibodies. B. γ2A_GHR_Jak2 cells were transfected with control siRNA (lanes 1 and 2), clathrin heavy chain siRNA (CHC) (lanes 3 and 4) or TrCP siRNA (lane 5 and 6). Cells were stimulated with 500 ng/ml GH for 90 min at 37°C as indicated, after which total cell lysates (TCL) (3 lower panels) and GHR immunoprecipitates (upper panel) were analysed on western blot with the indicated antibodies. C. γ2A_Jak2 cells, stably transfected with wild-type GHR (WT), GHR mutated in its DSGxxS motif (DSG), GHR mutated in its UbE motif (UbE) and GHR with all cytosolic tyrosine mutated into phenylalanine residues (Y-less) were stimulated with 500 ng/ml GH at 37°C as indicated, after which total cell lysates (TCL) (2 lower panels) and GHR immunoprecipitates (upper panel) were analysed on western blot with the indicated antibodies. Cell lines used in C are stably transfected with the indicated GHR constructs, resulting in clonal variation of Jak2 levels. Data in A and B are representatives of three independent experiments. Data in C are representatives of two independent experiments. D. Quantification of the GH-induced GHR degradation (Y-less from experiment in C, and the data from the experiment in B), expressed as percentage of mature GHR.

To compare GH-induced and GH-independent “constitutive” GHR endocytosis we used a γ2A, human fibroblast cell line that stably expresses GHR and is Jak2-deficient (γ2A_GHR). This cell line was used to make a variant cell line that produces low numbers of Jak2 (γ2A_GHR_Jak2). Upon stimulation of both cell lines with GH, quantification of mature GHR levels showed that after 90 min about 50% of the GHRs were degraded in γ2A_GHR_Jak2 cells, while in γ2A_GHR cells GHR steady state levels remained unchanged (Fig. 6A). These data indicate that GH is able to increase GHR endocytosis and lower the GHR steady state level only in the presence of Jak2. Without GH stimulation, the GHR steady state level in both cell lines is defined by constitutive GHR endocytosis, which results in much lower GHR levels in γ2A_GHR cells. We hypothesised that GH-induced as well as constitutive GHR endocytosis use the same endocytosis mechanism. If that is the case, GH-induced GHR endocytosis should (i) use the UbE motif and the SCF(βTrCP) ligase, (ii) depend on clathrin and (iii) not depend on the phosphorylation status of the GHR. The experiments of Fig. 3B already showed that both GH-induced and constitutive GHR endocytosis depend on the ubiquitin system. For the second criterion we asked whether GH-induced GHR endocytosis occurs via clathrin-coated pits. We depleted γ2A_GHR_Jak2 cells for clathrin, treated the cells with GH and compared the change in mature GHR levels between control and clathrin-depleted cells. Clathrin depletion strongly inhibited GH-induced GHR endocytosis, demonstrating that GH-induced GHR endocytosis is clathrin-dependent (Fig. 6B). Subsequently, we asked whether GH-induced GHR endocytosis depends on the same E3 ligase as constitutive GHR endocytosis. Depletion of βTrCP resulted in inhibition of GHR endocytosis for both constitutive and GH-induced GHR endocytosis (Fig. 6B). Quantification showed that GH-stimulated GHR endocytosis is reduced from 50% to about 20% under conditions of either clathrin or βTrCP depletion, indicating that both proteins are required for GH-induced GHR endocytosis.

Previous data indicated that βTrCP uses the non-canonical UbE motif for GHR endocytosis, although the canonical DSG motif is present in the GHR cytosolic tail. The question whether the DSG motif is involved in GH-induced endocytosis is relevant, because in case of Prl-induced PrlR degradation, the DSGxxS motif acts as endocytosis motif [43]. To clarify this point for the GHR we used either UbE or DSGxxS GHR mutant cell lines, derived from the γ2A cells expressing low numbers of Jak2. Upon GH treatment we observed that mutation of the UbE motif resulted in inhibition of GH-induced GHR endocytosis (Fig. 6C), indicating that the UbE motif is essential for GH-induced GHR endocytosis. Mutating the DSGxxS motif did not inhibit GH-induced GHR endocytosis (Fig. 6C). We conclude that both GH-induced GHR endocytosis and constitutive GHR endocytosis depend on βTrCP binding via the UbE-motif rather than via the DSGxxS motif. This implies that GH-triggered tyrosine phosphorylation of the GHR is irrelevant in our model. To explicitly address this issue we used our γ2A cell line expressing both low amounts of Jak2 and a mutant GHR in which all cytosolic tyrosine residues were replaced by phenylalanine (GHRYless). Fig. 6C and 6D confirmed that mutation of all tyrosines did not affect GH-induced GHR endocytosis.

In summary, we found that GH-induced GHR endocytosis and constitutive GHR endocytosis share the same characteristics. Both pathways are ubiquitin system-, clathrin- and βTrCP-dependent and use the UbE motif for GHR endocytosis. These data strongly suggest that the two pathways are similar.

### Relative high Jak2 levels compared to βTrCP correlate with high GH sensitivity of cells

**Figure 7:**
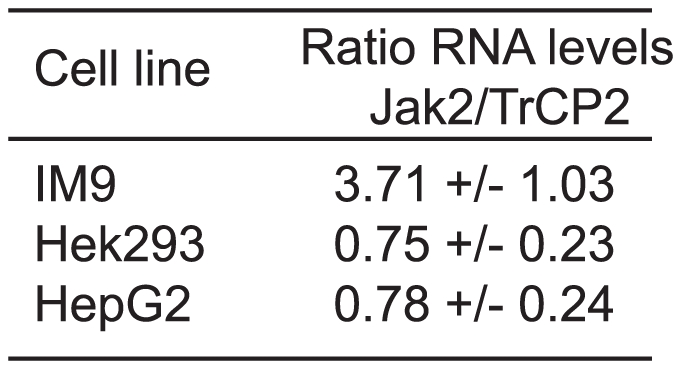
mRNA levels of Jak2 and βTrCP2 in IM9, Hek293 and HepG2 cells. mRNA levels were measured using qRT-PCR. Ratios of Jak2/βTrCP2 are displayed of IM9, Hek293 and HepG2 cells as mean values of six independent experiments ± SD.

The data presented in this study imply that both βTrCP2 and Jak2 bind to membrane-proximal linear motifs of the GHR at the cell surface. Therefore, relative expression levels of these two proteins might be important determinants for the endocytosis of GHR and possibly other cytokine receptors. If cells have relatively high Jak2 levels compared to βTrCP2, Jak2-dependent cytokine receptors will be stabilised at the plasma membrane. Conversely, relatively low Jak2 levels will result mainly in constitutive endocytosis. We tested three different cell lines for mRNA and protein levels of Jak2 and βTrCP2: IM9 cells that completely depend on GH for GHR endocytosis, and Hek293 and HepG2 cells that exhibit mainly constitutive endocytosis of exogenous GHRs. Using qRT-PCR we found that the Jak2 mRNA level relative to the βTrCP2 level is considerably higher in IM9 cells compared to Hek293 and HepG2 cells (Fig. 7). Although mRNA levels do not necessarily reflect protein levels, the data support our hypothesis that endogenous Jak2 protein levels contribute to the GH responsiveness of cells, not only because of their kinase potential but also their homeostatic effect.

## Discussion

Previous studies on cytokine receptors showed that Jak family members are able to stabilise GHR, Epo, G-CSF, and Tpo receptors in addition to the type I interferon α/β receptor (IFNAR1) and the oncostatin M receptor [4], [5], [9], [19], [24], [44]. For Epo receptor it has been shown that Jak2 binding in the ER induces proper folding and efficient trafficking to the cell surface [4]. Studies on Tpo receptor describe that Jak2 stabilises the mature Tpo receptor on the cell surface without affecting maturation kinetics [19]. For GHR, it has been shown that Jak2 enables efficient processing of precursor receptor to mature receptor [45]. Whereas Jak2 does seem to affect neither Tpo nor Epo receptor endocytosis, Jak2 clearly inhibits GHR endocytosis. The different effects of Jaks on cytokine receptors suggest that cytokine receptors contain unique features in their cytoplasmic tails to be differentially affected. Whether a common theme will emerge, i.e. that the kinases stabilise their cognate receptors until receptor activation, remains to be investigated. In summary, the data of our study indicate the following scenario: Jak2 binding prevents endocytosis until GH binding triggers Jak2 phosphorylation and its detachment from the GHR, presumably via the phosphorylation of Y119 in Jak2. GHRs, synthesised in excess to uncommitted Jak2, are continuously and βTrCP-mediated endocytosed. Thus, the GHR depends on GH for endocytosis only if Jak2 is bound. This is in agreement with the study of Deng *et al.*, who showed that at low concentration of Jak2 GHR degradation depends on GHR activation [24].

For the IFNARI subunit as well as for PrlR ligand-induced phosphorylation of a DSGxxS motif C-terminal of box-2 triggers endocytosis and degradation [43], [46]. Although such a conserved motif is also present in GHR, we showed that it is not involved in GH-induced endocytosis. The present study demonstrates that the UbE motif is essential for both GH-induced and constitutive GHR endocytosis. In addition, we find that the tyrosine residues of the GHR are not involved in GHR endocytosis. This part is in contrast to data by Deng *et al.* [24], who showed that phosphorylation of cytosolic tyrosine residues is important for GH-induced GHR endocytosis. Since the expression level of Jak2 is essential to be able to measure GHR endocytosis, a different Jak2 expression level in our model system compared to the model system of Deng *et al.* could explain the different findings.

In our proposed model, the release of phosphorylated Jak2 from the GHR is induced by GH stimulation, after which βTrCP can bind the GHR and select the receptor for endocytosis. This implies that the role of Jak2 is upstream of βTrCP binding and subsequent GHR endocytosis. However, the Jak2 release and subsequent GHR endocytosis cannot be measured in cells overexpressing GHR, because the level of exogenous GHR greatly exceeds the number of endogenous Jak2. As a consequence, the vast majority of GHRs is not in complex with Jak2 and will endocytose constitutively (independent of Jak2). In our previous studies of Alves dos Santos *et al.*. cellular model systems were used in which GHR was overexpressed [47], [48]. Therefore, the authors concluded that Jak2-binding does not induce GHR endocytosis, but it cannot be excluded that Jak2 has an inhibitory effect on GHR endocytosis. The same arguments apply for the observation that endocytosis of GHRs that lack a functional box-1 is unaffected. Moreover, Alves dos Santos *et al.* have shown Jak2 binding in the endosomes, suggesting that Jak2 remains associated with the GHR during the process of endocytosis. However, it is well possible that this (small) amount of Jak2 has rebound after GHR endocytosis.

Our conclusion that Jak2 binding inhibits GHR ubiquitination and probably acts upstream of SCF(βTrCP) and clathrin is based on the K48 ubiquitination status of the GHR. If Jak2 detaches, the activity of SCF(βTrCP) seems to increase due to GH-induced Jak2 phosphorylation. When Jak2(1-525) is over-expressed, no GH-induced increased ubiquitination of the receptor occurs. This could be justified by the fact that Jak2(1-525) lacks kinase activity and, therefore, does not phosphorylate itself and consequently does not detach. Overexpression of the Y119E mutant doesn't affect the GH-induced GHR increased ubiquitination since it does not bind. Together, these date clearly support our model.

In addition to phosphorylation of Jak2, cells must have other mechanisms available to respond to exogenous factors that also induce Jak2 detachment in order to decrease GH sensitivity. These scenarios are required when stresses impose survival mechanisms. In these cases, stress-driven signal transduction pathways might activate Jak2 to abrogate the GHR-Jak2 interaction and induce rapid endocytosis via SCF(βTrCP). Alternatively, a protein that interferes with the Jak2-GHR interaction could be up regulated. No such factor has been identified for the Jak2-GHR interaction yet.

Our findings implicate that, in addition to the expression level of GHR, the cellular concentration of free Jak2 is a major factor that regulates the number of GHRs at the cell surface. A simple explanation would be that the N-terminal half of Jak2, bound to box-1, is sufficient to shield other functional motifs like the UbE sequence, or prevent SCF(βTrCP) complex assembly. Evidence for this scenario comes from a study from Pelletier *et al.*, in which they show that both box-1 and the UbE-motif are involved in Jak2 binding and activation [33]. The question whether both the UbE and the box-1 positions can be occupied at the same time, by βTrCP and Jak2 respectively, remains unanswered. The stoichiometry of a kinase-competent complex requires the presence of two Jak2 molecules, while an endocytosis-competent complex probably requires two complete SCF(βTrCP) complexes with two neddylated cullins. βTrCP acts as a dimer and possibly needs two UbE motifs for productive binding. On the other hand, it might well be that one Jak2 molecule on a GHR dimer is sufficient to block endocytosis [17], [49]. Our current experiments indicate that both Jak2 and βTrCP can be present at the same time, since overexpression of Jak2 did not affect βTrCP binding to the GHR, in pull-down experiments with biotinylated GH (not shown). However, as suggested by the BRET experiments, the system appears highly dynamic. As endocytosis of monomeric GHR does not require the ubiquitin system [17], only *in vitro* binding studies with dimeric GHRs will elucidate the interplay between Jak2 and SCF(βTrCP).

As many genes have sexually dimorphic expression dependent on either male pulsatile or female continuous patterns of GH secretion [50], our model predicts that cellular Jak2 levels might contribute to this; cells with high Jak2 levels and low GHR expression, like IM9, respond mainly to GH spikes, because they lose their GHRs temporarily, while liver cells with low Jak2 levels and high GHR expression are continuously responsive for both types of GH secretory patterns.

## Materials and Methods

### Materials, antibodies and DNA constructs

MβCD and staurosporin were purchased from Sigma. MG132 was obtained from Calbiochem. GHR antisera were described before [14]. Monoclonal 4G10 anti-pY was obtained from Upstate (Millipore). Monoclonal antibodies against Jak2 (AHO1352), ubiquitin (clone FK2) and clathrin (C1860) were bought from Biosource, Biomol and Sigma, respectively. Anti-STAT5 (C17) was obtained from Santa Cruz Biotechnologies Inc. and anti-actin (Clone C4) was obtained from MP Biomedicals Inc. Monoclonal anti-Flag (M2) was from Sigma. Polyclonal anti-Jak2 was described previously [34] and anti-βTrCP1 was described before [16]. Full-length rabbit GHR cDNA in pcDNA3 has been described before [14], just as GHR(S366A, S370A) (DSG mutant) [16]. The Flag- tagged wild-type mouse Jak2 constructs were a generous gift from Prof. Carter-Su (University of Michigan, Ann Arbor). Flag-Jak2(1-525) was constructed by the introduction of a stop-codon using the Quickchange mutagenesis kit from Stratagene. Jak2(Y119E), the GHR in which all tyrosines were mutated to phenylalanines and the GHR in which 326EFIxxD residues were mutated to alanine (UbE mutant), were also produced using the same kit. Protein A-beads were from Repligen. We cloned Jak2 into vector pSG213 for stable expression in γ2A cells. pSG213 was a kind gift of Prof. Melchior (Zentrum für Molekulare Biologie der Universität Heidelberg).

### BRET contructs

Renilla Luciferase (Rluc) expression vector was a gift from Michel Bouvier (Université de Montrèal). The primers were purchased from Sigma-Genosys. We amplified the Rluc coding region with the forward primer GATCGCGGCCGCATGACTTCGAAAGTTTATG and the reverse primer GATCTCTAGATTATTGTTCATTTTTGAGAACTCG. The Rluc-pcDNA3 construct was generated by ligating the Rluc fragment into pcDNA3 (Invitrogen) using Not1 and Xba1 restriction sites. GHR cDNA was amplified from GHR-pcDNA3 expression vector [14] with the forward primer, GATCGGTACCGCCACCATGGATCTCTGGCAG and the reverse primer GATCCCAGTGTGCTGGTGGCAAGATTTTGTTCAG. The obtained PCR product was cloned in frame with the Rluc coding sequence in the previously generated Rluc-pcDNA3 vector, using Kpn1 and BstX1 restriction sites. Expression from this vector, GHR-Rluc-pcDNA3, results in C-terminally Rluc tagged GHR. The YFP sequence was amplified from EYFP-N1 vector (Clontech), using the forward primer GATCCTTAAGGCCACCATGGTGAGCAAG, and the reverse primer, GATCGGTACCCTTGTACAGCTCGTCCATGCC and the PCR product was cloned into the doxycycline inducible promotor pcDNA4/TO (Invitrogen) using Afl2 and Kpn1 restriction sites. Jak2 (wt) and Jak2 (Y119E) were amplified from pcDNA3-flag-Jak2(wt) and pcDNA3-Flag-Jak2 (Y119E) expression vectors, respectively, using the forward primer GATCGGATCCATGGGAAATGGCCTGCC and reverse primer GATCGCGGCCGCCTATACTGTCCCGGATTTGATCC. The obtained PCR products were cloned in frame with the YFP coding sequence in the previously generated YFP-pcDNA4 vector, using the restriction sites BamH1 and Not1. Expression from these vectors, YFP-Jak2(wt or Y119E)-pcDNA4, results in N-terminally YFP tagged Jak2.

### Cell culture, transfections, and microscopy

Human embryonic kidney 293 (Hek293) cells, stably expressing wtGHR were maintained in DMEM high glucose (4.5 g/l), supplemented with 10% FCS, 100 U/ml penicillin, 100 µg/ml streptomycin and 600 µg/ml G418 as described previously [16]. IM9 cells were maintained in RPMI 1640, 10% FCS, penicillin, streptomycin, containing 4.5 g/l glucose and 1 mM sodium pyruvate. γ2A cells were maintained in DMEM low glucose (1.0 g/l), supplemented with 10% FCS, 100 U/ml penicillin, 100 µg/ml streptomycin. Stable cell lines with Jak2 in pSG213 were maintained in medium, supplemented with puromycin (1 µg/ml), and stable cell lines with wtGHR with hygromycin 50 µg/ml). When indicated, the cells were pretreated with 10 mM MβCD, 20 µM MG132 or staurosporin in serum free medium for 60 min at 37°C. Human GH was added at a concentration of 180 ng/ml. DNA transfections were performed using FuGene 6 (Roche, Applied Sciences) according to the manufacturer's instructions. Clathrin was silenced using the ON-TARGETplus SMART-pool of Dharmacon (Thermo Fisher Scientific., Lafayette, CO). βTrCP (1 and 2) was silenced with the “combi probe” and GFP siRNA was used as described before [16]. In all siRNA transfections Lipofectamine 2000 (Invitrogen) was used according to the manufacturer's instructions. Microscopy studies were performed as previously described [16].

### Generation of stable cell lines for the BRET studies

Hek293 cells, stably expressing the Tetracycline Repressor (Hek293-TR), were a gift from Dr. Madelon Maurice (Dept Cell Biology, UMC Utrecht). The cells were transfected with GHR-Rluc-pcDNA3 construct using Fugene 6 (Roche, Applied Sciences), according to the manufacturer's instructions. The selection of clones expressing GHR-Rluc was done using Geneticin (Invitrogen). Subsequently, double stable cell lines expressing GHR-Rluc and YFP-Jak2 (wt or Y119E) were generated, by transfecting YFP-Jak2 (wt or Y119E)-pcDNA4 in the previously generated cell line expressing GHR-Rluc. The selection of clones expressing GHR-Rluc and YFP-Jak2 (wt or Y119E) was done using zeocin (Invitrogen). The generated BRET cell lines were maintained in DMEM high glucose (4.5 g/l) (Invitrogen), supplemented with 10% FCS (Invitrogen), 100 U/ml penicillin (Invitrogen), 100 µg/ml streptomycin (Invitrogen), 600 µg/ml Geneticin (Invitrogen) and 100 µg/ml Zeocin (Invitrogen). The expression of YFP-Jak2 was induced by addition of 1 µg/ml doxycycline (Clontech) 24 h before the experiment.

### Lysis and immunoprecipitations

For GHR-Jak2 co-immunoprecipitations, the cells were lysed in 20 mM Tris pH 8.0, 150 mM NaCl, 0.5% NP40, 1 mM PMSF, 10 µg/ml aprotinin, 10 µg/ml leupeptin. For phosphorylation and ubiquitination experiments, the cells were lysed in 1% Triton X-100 with inhibitors (1 mM EDTA, 1 mM PMSF, 10 µg/ml aprotinin, 10 µg/ml leupeptin, 10 mM NEM, 1 mM Na_3_VO_4_ and 50 mM NaF). To prevent incomplete solubilisation or association of ubiquitinated proteins for some experiments cells were lysed in hot SDS sample buffer containing 1% SDS. Cell lysates were centrifuged to pellet the nuclei and the supernatants were used for GHR isolation in 1% Triton X-100, 0.5% SDS, 0.25% sodium deoxycholate, 0.5% BSA, and inhibitors via immune precipitation with anti-GHR and protein A beads. Immunoprecipitates were subjected to reducing SDS-PAGE and transferred to Immobilon-FL polyvinylidenedifluoride membrane (Millipore). Blots were immunostained with the indicated primary antibodies followed by Alexa Fluor 680, Alexa-800 IRDye conjugated anti-mouse or anti-rabbit antibodies. Detection was performed with an Odyssey system (LI-COR Biosciences).

### ^125^I-GH binding and internalisation and cell fractionation

^125^I-GH binding and internalisation was performed as previously described [15]. Internalisation was expressed as a ratio between iodinated GH inside and total. For cell fractionation, 10^7^ (IM9) cells were incubated for 10 min with GH and washed with cold PBS. Cell fractionation was performed as previously described [32], except that we omitted the 12.500g centrifugation step. After fractionation, the membrane and cytoplasmic fractions were immunoprecipitated with polyclonal anti-Jak2, followed by isolation using protein A beads. The samples were subjected to gel electrophoresis and western blotting as described above.

### BRET measurements

24 h prior to the measurements, cells were washed with phosphate-buffered saline (PBS), and detached with trypsin-EDTA. 4×10^4^ cells were reseeded in DMEM phenol-red free medium (Gibco), containing 10% FCS, 100 U/ml penicillin, 100 µg/ml streptomycin per well of a 96-well plate (white cell culture plate Nunc), in the presence or absence of doxycycline. 2×10^5^ cells treated in the same way were seeded per well in 24 wells plates and lysed 24 h after to evaluate the expression levels of the BRET pairs. Before the BRET measurements the culture medium was replaced by BRET assay buffer: PBS, 0,1% glucose, 25 mM Hepes. The substrate ViviRen (Promega) was then added to each well in a final concentration of 60 µM. 30 sec before the measurement started, 200 ng/ml hGH was added for the indicated times points. Repeated readings at 475 nm and 535 nm (15 cycles, 0,5 sec interval time, 15 sec per reading), for donor and acceptor emission, respectively, were taken at 37°C with a Fluorstar Optima Fluorescence Plate Reader (BMG LABTECH). BRET signal was expressed in milliBRET Units as defined previously [39]. The BRET unit is the signal ratio 535/475 nm obtained when the donor and acceptor are expressed (in presence of doxycycline), subtracted from the ratio obtained under the same experimental conditions, when only the donor partner (fused to Rluc) is expressed (in absence of doxycycline). Two ANOVA analyses of variance were performed with the statistical program SPSS where the factors were compared in the presence/absence of GH and at the time periods after GH addition.

### RNA isolation and real time quantitative–PCR (qRT-PCR)

Total RNA from IM9, Hek293 and HepG2 cells was isolated using RNeasy mini kit (Qiagen) according to the manufacturer's protocol. Synthesis of cDNA was carried out from 1 µg total RNA in 20 µl reaction volumes using the iScript™ cDNA synthesis kit according to the manufacturer's protocol (BioRad). Primers were designed using primer select software of DNA star (Madison, WI) according to the parameters outlined in the BioRad i-cycler manual. The specificity of each primer was confirmed by sequencing its product. Rps19 and β-actin genes were used as the non-regulated reference genes for normalisation of target gene expression. Sequences of the used primers are for Jak2 TGAAGACCGGGATCCTACACACAGTT (forward) and GTCATACCGGCACATCTCCACAC (reverse), for βTrCP2 CATGTTGCAGCGGGACTTTATTACC (forward) and GATCACTCGCTGCCATTCTTTACAT (reverse), for Rps19 CCTTCCTCAAAAAGTCTGGG (forward) and GTTCTCATCGTAGGGAGCAAG (reverse), for β-actin. TCCCTGGAGAAGAGCTACG (forward) and GTAGTTTCGTGGATGCCACA (reverse). Annealing temperatures for Jak2, βTrCP2, Rsp19 and β-actin were 63.0, 62.0, 61.0 and 60.0°C, respectively. QRT-PCR was performed using BioRad MyIQ detection system (BioRad) with SYBR green fluorophore. Data analysis was carried out using the pair wise fixed reallocation and randomisation test incorporated in the software program REST-MCS [51] at 5% level of significance. Experiments were performed in duplicate and values from six experiments were used for data analysis.
